# An Insight into Internal Resorption

**DOI:** 10.1155/2014/759326

**Published:** 2014-05-12

**Authors:** Priya Thomas, Rekha Krishna Pillai, Bindhu Pushparajan Ramakrishnan, Jayanthi Palani

**Affiliations:** Department of Oral and Maxillofacial Pathology, Annoor Dental College & Hospital, Muvattupuzha, Ernakulam District, Kerala 686673, India

## Abstract

Internal resorption, a rare phenomenon, has been a quandary from the standpoints of both its diagnosis and treatment. It is usually asymptomatic and discovered by chance on routine radiographic examinations or by a classic clinical sign, “pink spot” in the crown. This paper emphasizes the etiology and pathophysiologic mechanisms involved in internal root resorption. Prognosis is good for smaller lesions; however, for those with extensive resorption associated with perforation the tooth structure is greatly weakened and the prognosis remains poor.

## 1. Introduction

Tooth resorption presents itself either as a physiological or a pathological process occurring internally (pulpally derived) or externally (periodontally derived). According to the Glossary of the American Association of Endodontists, resorption is defined as a condition associated with either a physiologic or a pathologic process resulting in the loss of dentin, cementum, or bone [1].

External resorption begins from the external or cervical surface of the tooth and proceeds inwards and is associated with factors like periapical pathosis, pressure from orthodontic treatment, and rapidly growing tumors. Internal resorption (IR) is a rare, insidious, resorptive pathological process, beginning in the pulpal space and extending into the surrounding dentin. Its diagnosis and management have been a challenge to dental practitioners. Due to its insidious pathology, internal resorption can progress to a great extent before its detection. Treatment is by arresting the cellular activity accounting for the resorptive process.

## 2. Classification: SeeTable 1

Resorption is of various types. Physiologic resorption is associated with deciduous dentition resulting in their exfoliation, paving the pathway for the eruption of their succedaneous teeth. Unlike the deciduous teeth, the permanent teeth rarely undergo resorption unless stimulated by a pathological process. Pathologic resorption occurs following traumatic injuries, orthodontic tooth movement, or chronic infections of the pulp or periodontal structures [1]. If the process is left untreated, it will result in the premature loss of the affected teeth [2].

Internal resorption is noticed in the inner walls of root canal while external resorption is noticed on the root surface or cervical area. External root resorption exists concurrently with resorption of the alveolar bone and the resorptive process presents in a similar manner to that of bone.

## 3. Pink Tooth of Mummery/Internal Resorption

Internal resorption was first reported by Bell in 1830. Pink tooth of Mummery (1920), so called due to the presence of a pink discoloration on the crown, is named after the anatomist Mummery [3].

Internal resorption can be categorized by the type/cause of resorption as inflammatory, transient, progressive, and replacement [1] (seeTable 2). Inflammatory resorption is more likely to commence following damage to the predentin subsequent to a bacterial infection or trauma [4]. A transient internal resorption is another form of trauma induced noninfective, self-limiting root resorption and this will generally follow a luxation injury [5, 6]. Progressive type of internal resorption is stimulated by constant inflammation following infection [7]. Root canal replacement/metaplastic resorption involves subsequent deposition of mineralised tissue resembling bone or cementum but not dentin [1]. Reichart et al. [8] entitled this metaplastic tissue as “reparative cementum” or “osteodentin-like material.”

The prevalence, etiology, and natural history of internal resorption are uncertain [7] and the associated key molecular pathogenesis/events have not been understood completely.

Dentin is lined internally from the pulpal surface by the odontoblastic layer and predentin. The two layers together form a barrier and prevent its resorption. Many studies have proven that, similar to the osteoclasts, the odontoclasts do not adhere to or resorb unmineralized matrix [9]. Wedenberg and Lindskog reported that damage to both these layers result in exposure of the underlying mineralized dentin to odontoclasts making it vulnerable to resorption [9]. The uncommon occurrence of dentin resorption can furthermore be explained by the dominance of odontoclast inhibitory substances such as OPG (osteoprotegerin) over activators like RANKL (receptor activator of nuclear factor kappa B ligand) [5]. It has also been suggested that dentin contains a noncollagenous component which may function as a resorption inhibitor [5].

Internal inflammatory resorption commence following a stimulus (inflammation) with the elimination of inhibitory mechanism, that is, the loss or alteration of the protective (predentin/odontoblastic) layer and pulpal invasion by multinuclear giant cells/odontoclasts/dentinoclasts [10, 11]. These giant cells are morphologically similar to osteoclasts, with related enzymatic properties and resorption patterns. They are probably derived from tartrate resistant acid phosphatase (TRAP) positive circulating monocytes [12]. However, odontoclasts are smaller in dimension, with fewer numbers of nuclei, and are present within smaller resorption lacunae [1, 13]. They do not show the well-developed clear zones of active osteoclasts. This has been attributed to the difference in composition of the dental tissues and bone [14].

### 3.1. Etiology

It has been documented that the process is initiated by a variety of stimuli such as trauma, pulpotomy, extreme heat produced during cutting of dentin [15], chronic inflammation of the pulp following caries perpetuated by bacterial factors, cracked tooth, tooth transplantation, and orthodontic treatment [4, 10, 16].

The literature also cites the association of herpes zoster with resorption and the degeneration of odontoblast due to systemic viral infection [17]. Solomon et al. [16] reported a case of internal resorption affecting 21 and 23, with a history of herpes zoster affecting the maxillary branch of the left trigeminal nerve. The effect on the dental pulp could be possibly explained by the virus being attracted to the nerve endings in the pulp [18]. Ramchandani and Mellor [18] and Wadden [19] have also accounted cases of internal resorption linked with herpes zoster infection.

Genetic factors have also been implicated in the development of internal resorption. The link between interleukin- (IL-) 1 gene polymorphism and root resorption has been reported in a study of monozygotic twins [20].

### 3.2. Pathophysiology: See Figures 1 and 2

Internal resorption usually occurs as a result of a continuous chronic inflammatory process. Progress of internal resorption is dependent on two things: presence of vital pulp tissue at/below the resorption area and partially or completely necrotic pulp, coronal to the site of resorption, thereby allowing a constant entry of microorganisms and its antigens into the root canal. Microbial stimulus is an essential factor for the persistence of resorption [5]. The extent of progression is also determined by the intensity of the stimuli and inflammatory process. The origin of clastic cells is related to the viable blood supply and the necrotic tissue acts a stimulus for the formation of these cells. This probably explains as to why IR is stated as a rare occurrence compared to external root resorption [21]. The vascular changes in the pulp produce hyperaemia, causing an increased oxygen tension resulting in low pH levels, thus attracting numerous macrophages to the site, thereby piloting the onset of resorptive process [22]. The connective tissue, following the resorptive activity, may undergo metaplasia to form granulation tissue [22]. Predominance of a progressing infection causes necrosis of the entire pulp tissue and limits the resorptive process and this acts as a protective mechanism preventing its progression [5]. The presence of a collateral blood supply through an accessory canal from the periodontal ligament to the resorption site can add to maintaining the resorptive process. Internal inflammatory root resorption in its most classical form spreads symmetrically in all directions into the dentin surrounding the pulp [5].

Replacement type of internal resorption results from a low grade irritation of pulpal tissues such as chronic irreversible pulpitis or partial necrosis usually localized to a small area in the root canal [23]. This associated low grade chronic infection produces more of a reactive lesion bringing about deposition of metaplastic tissue resembling bone or cementum [24]. Wedenberg and Zetterqvist examined cases of progressive internal resorption and observed that the normal pulp tissue was replaced by a periodontal-like connective tissue with osteogenic potential. Furthermore, the process appeared to alternate between resorption of dentin and apposition of mineralized tissue, the extent of the former being related to the intensity of stimuli [25].

Transient apical resorption may follow luxation injuries and this can be viewed through serial X-rays showing a reduction in radiolucency over a period of a few months.It is an affirmative response, as the traumatized pulp heals rapidly aided by the presence of a viable vascular network. An associated colour change may evolve due to intrapulpal haemorrhage and might resolve instinctively upon the revascularization of the coronal pulp chamber. As this is a transient process, the internally resorbed apex will resolve uneventfully [26].

## 4. Clinical Characteristics

Internal resorption is usually asymptomatic and approximately only 2% shows clinical signs [27]. It is more frequently observed in males than females [28]. Haapasalo suggested a prevalence of 0.01%–1% for internal root resorption occurring due to inflammatory causes [5]. Wedenberg and Zetterqvist reported that there was no clinical or morphological difference in internal resorption between primary and permanent teeth, although the resorption progressed more rapidly in primary teeth [25]. Internal resorption is usually found either in the mid or apical area of root. They are discovered by chance on routine radiographs or by the clinical sign of a “pink spot” on the crown. The pulp can either show partial or complete necrosis. In an actively progressing lesion, the tooth may be partially vital and may present symptoms typical of pulpitis [5]. The coronal portion of the pulp is often necrotic, whereas the apical pulp, which includes the internal resorptive defect, can remain vital [10]. Pain may be a presenting symptom if there is complete perforation of the crown and the granulation tissue is exposed to oral environment.

A pinkish color, the “pink tooth,” is generated, in which the resorptive process has extended to the cervical area of the crown, consequential to the granulation tissue ingrowths [5, 7]. Another characteristic feature that gives an indication about the centre of origin of the resorption is the location of “the spot”: a color change from inflammatory internal resorption seen typically in the middle of the tooth in the mesiodistal direction (except in multirooted teeth), whereas in cervical resorption it is located either mesially, centrally, or distally [5].

Progressive resorptions can result in extensive unrestorable tissue loss. At times an external communication can develop between the root canal and the periodontal ligament, due to perforation of the root.

## 5. Radiological Features: SeeFigure 3

The diagnosis of internal resorption is mainly based on radiographs. A considerable amount of pulpal dentin wall must be resorbed to be reliably detected in the radiograph. In cases where the infection spreads rapidly through the root canal resulting in necrosis of the entire pulp, the resorption terminates at an early stage and remains undetected both clinically and radiographically [5]. Infection induced/inflammatory IR is detected as round to oval shaped radiolucencies contained within the root canal along with symmetrical widening of canal space. The classical radiographic description of internal resorption is outlined by Gartner et al. as a clearly well-defined symmetrical radiolucency of uniform density which balloons out of the pulp chamber or root canal [29].

In single-rooted teeth with one root canal, the internal resorption starts out as a symmetrical lesion in coronal pulp/crown area. However, in multirooted teeth with a wide pulp chamber, internal resorption begins at one part of the chamber and spreads locally into the surrounding dentin. Radiographically internal resorption occurring apically may be difficult to diagnose when the resorptions are of the lower grades as described by Vier and Figueiredo [30]. Diagnosis becomes a major challenge for resorptive defects on the facial/lingual/palatal aspects, as they are often missed during examination. Newer radiographic techniques, such as tuned aperture computed tomography (TACT), display significant promise to identify these defects [31]. Another diagnostic sign is the manner in which the pulp “disappears” into the lesion, not extending through the lesion in its regular shape. Lynch and Ahlberg reported a bilateral idiopathic internal resorption where the radiolucent areas were clearly defined, punched-out lesions with the pulp seeming to disappear into the lesion [32].

## 6. Histopathology

An internal resorption lesion mainly consists of granulation tissue. The pulpal connective tissue is highly vascularized with varying degrees of inflammation, infiltrated by lymphocytes, macrophages, neutrophilic leukocytes, andplasma cells. Neutrophils and macrophages are attached to the mineralized dentin surface. “Resorptive bays” with numerous odontoclasts are also seen [25]. Bacteria can be detected either in the dentinal tubules or in the necrotic part of the coronal root canal or in dentinal tubules communicating between the necrotic zone and the granulation tissue of teeth undergoing rapidly progressing resorption. Allen and Gutmann reported osteoid or cementum-like tissue in some areas of the pulpal wall as well as small calcifications in the pulp tissue [33].

## 7. Diagnosis and Differential Diagnosis of Internal Resorption

There are certain key factors that may be looked into for the diagnosis of internal resorption.

### 7.1. Etiology

History of trauma, other etiological factors like crown preparation or pulpotomy, and positive pulp sensitivity test should be ruled out for its diagnosis.

### 7.2. Sensitivity Testing

A negative response to sensitivity testing is obtained, as the coronal pulp has often been removed or is necrotic and the active resorbing cells are more apical in the canal.

### 7.3. Pink Spot

The pink tooth caused by the granulation tissue undermining the enamel can also be a feature of subepithelial external inflammatory root resorption/cervical resorption (progressive external root resorption, of inflammatory origin, occurring immediately below the epithelial attachment of the tooth), which must be ruled out before the diagnosis of internal resorption is established [10].

### 7.4. Radiology

Progressive internal resorption or those with perforations of root can be distinguished from external resorption by varied radiographic techniques. In teeth with internal resorption, the radiolucent lesion “moves” with the canal when the radiographs are taken at different angles, while in external resorption the radiolucent lesion “moves” outside of the canal [34]. Internal resorption has a uniform enlargement of the canal space with regular bone structure, whereas external resorption has an irregular border with an alteration in the adjacent bone, and the canal can often be visualized through the radiolucent area [34].

The majority of misdiagnosis of resorptive defects is made between subepithelial external and internal resorption. If root canal therapy is opted for an evident internal resorption, bleeding within the canal should cease quickly on pulp extirpation, as the source of blood supply is from the granulation tissue and apical blood vessels. If bleeding continues during treatment and presents even at the second visit, the source of the blood supply is external and treatment for external resorption should be carried out. Any continuation of theresorptive process on recall radiographs even after an effective root canal therapy suggests the possibility of an external resorptivedefect being misdiagnosed [10].

## 8. Treatment and Management

Delayed treatment can lead to progressive internal resorption. However, it can be arrested in its initial phase if detected earlier. The prognosis of treatment depends on the extent or the size of lesion. When internal resorption is clinically detected, pulpectomy is needed to arrest the resorption. Calcium hydroxide has been known to be a potent inhibitor of inflammation. When resorption has progressed to involve an external communication, the tooth cannot be retained in most cases. In some situations, complex surgical procedures need to be considered to gain access for repair [35].

According to Culbreath et al., several materials such as gutta-percha, zinc oxide eugenol, and amalgam alloy can be used for the treatment for internal resorption [36]. These materials do not provide sufficient strength to the tooth structure and may also cause substantial tooth discoloration. Endodontic treatment should be attempted within 7–10 days of the injury, in teeth with closed apices, as the revascularization cannot occur, before the necrosed pulp becomes infected [35]. Currently mineral trioxide aggregate is being increasingly used as a root canal filling material in these perforations [37]. In teeth with a large resorption cavity in the coronal third of the root canal, use of composite materials should be considered in order to strengthen the tooth and to make it more resistant to tooth fracture [38].

## 9. Conclusion

Internal resorption is a rare insidious process with the majority of the cases being idiopathic. The detection of internal resorption is easy only in simple cases, while others require high quality periapical radiographs or advanced diagnostic techniques like dental CT scans for its diagnosis. Proper patient history, early diagnosis, and appropriate treatment at the correct time prevent tooth loss. The outcome of the treatment is good and depends on the amount of remaining dentin wall thickness. Care should be taken to distinguish internal resorption from other types of tooth resorption for appropriate management.

## Figures and Tables

**Figure 1:**
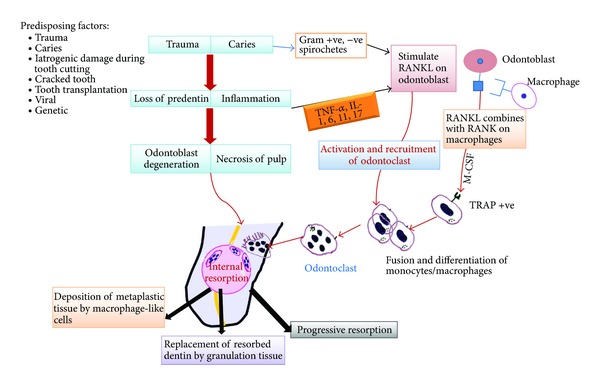
Schematic representation of pathogenesis of internal resorption.

**Figure 2:**
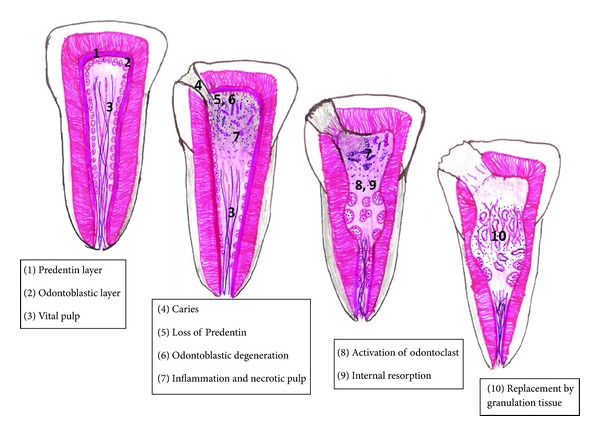
Diagrammatic representation of pathogenesis of internal resorption.

**Figure 3:**
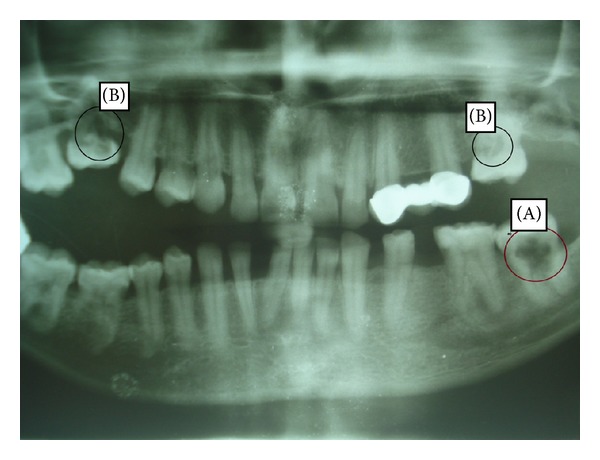
OPG showing multiple resorptions, (A) internal resorption of 37 and (B) cervical resorption.

**Table 1:** Classification based on type of process, location, and the type and cause.

Type of process	Location in relation to root surface	Type/cause of resorption
(i) Physiological resorption (ii) Pathological resorption	(i) Internal (ii) External/cervical	(i) Inflammatory (ii) Transient (iii) Progressive (iv) Replacement

**Table 2:** Modified Andreasen classification.

Modified Andreasen classification based on the type of resorption	
Internal resorption	(a) Internal inflammatory resorption
	(b) Internal replacement resorption
	(c) Transient apical breakdown

External resorption	(a) External surface resorption
	(b) External inflammatory resorption
	(c) External replacement resorption
	(d) External cervical resorption
	(e) Transient apical breakdown

## References

[1] Ne RF, Witherspoon DE, Gutmann JL (1999). Tooth resorption. *Quintessence International*.

[2] Patel S, Ricucci D, Durak C, Tay F (2010). Internal root resorption: a review. *Journal of Endodontics*.

[3] Mummery JH (1920). The pathology of “ pink-spots” on teeth. *British Dental Journal*.

[4] Tronstad L (1988). Root resorption—etiology, terminology and clinical manifestations. *Dental Traumatology*.

[5] Haapasalo M, Endal U (2006). Internal inflammatory root resorption: the unknown resorption of the tooth. *Endodontic Topics*.

[6] Andreasen FM (1986). Transient apical breakdown and its relation to color and sensibility changes after luxation injuries to teeth. *Dental Traumatology*.

[7] Holmes JP, Gulabivala K, van der Stelt PF (2001). Detection of simulated internal tooth resorption using conventional radiography and subtraction imaging. *Dentomaxillofacial Radiology*.

[8] Reichart PA, Dürr U-M, Triadan H, Vickendey G (1984). Periodontal disease in the domestic cat. A histopathologic study. *Journal of Periodontal Research*.

[9] Wedenberg C, Lindskog S (1987). Evidence for a resorption inhibitor in dentin. *Scandinavian Journal of Dental Research*.

[10] Trope M (2002). Root resorption due to dental trauma. *Endodontic Topics*.

[11] Pindborg JJ (1970). *Pathology of the Dental Hard Tissues*.

[12] Bhaskar SN (1986). *Orban’s Oral histology and Embryology*.

[13] Harokopakis-Hajishengallis E (2007). Physiologic root resorption in primary teeth: molecular and histological events. *Journal of Oral Science*.

[14] Hammarström L, Lindskog S (1985). General morphological aspects of resorption of teeth and alveolar bone. *International Endodontic Journal*.

[15] Gottlieb B, Orban B (1930). Veranderunngen in Periodontium nach chirurgischer Diathermie. *Zeitschrift für Stomatologie*.

[16] Solomon CS, Coffiner MO, Chalfin HE (1986). Herpes zoster revisited: implicated in root resorption. *Journal of Endodontics*.

[17] Seltzer S, Bender I (1975). *The Dental Pulp*.

[18] Ramchandani PL, Mellor TK (2007). Herpes zoster associated with tooth resorption and periapical lesions. *British Journal of Oral and Maxillofacial Surgery*.

[19] Wadden JV (1991). Extensive endodontic involvements following herpes zoster attack to facial area; report of a case. *Northwest Dentistry*.

[20] Urban D, Mincik J (2010). Monozygotic twins with idiopathic internal root resorption: a case report. *Australian Endodontic Journal*.

[21] Levin L, Trope M, Hargreaves KM, Goodis HE (2002). Root resorption. *Seltzer and Bender’s Dental Pulp*.

[22] Sikri VK (2011). Root resorption an enigma. *Indian Journal of Comprehensive Dental Care*.

[23] Benenati FW (1997). Root resorption: types and treatment. *General Dentistry*.

[24] Lindskog S, Blomlöf L, Hammarström L (1983). Repair of periodontal tissues *in vivo* and *in vitro*. *Journal of Clinical Periodontology*.

[25] Wedenberg C, Zetterqvist L (1987). Internal resorption in human teeth—a histological, scanning electron microscopic, and enzyme histochemical study. *Journal of Endodontics*.

[26] Heithersay GS (2007). Management of tooth resorption. *Australian Dental Journal*.

[27] Andreasen JO (1970). Luxation of permanent teeth due to trauma. A clinical and radiographic follow-up study of 189 injured teeth. *European Journal of Oral Sciences*.

[28] Goultschin J, Nitzan D, Azaz B (1982). Root resorption: review and discussion. *Oral Surgery, Oral Medicine, Oral Pathology*.

[29] Gartner AH, Mack T, Somerlott RG, Walsh LC (1976). Differential diagnosis of internal and external root resorption. *Journal of Endodontics*.

[30] Vier FV, Figueiredo JAP (2004). Internal apical resorption and its correlation with the type of apical lesion. *International Endodontic Journal*.

[31] Nance RS, Tyndall D, Levin LG, Trope M (2000). Diagnosis of external root resorption using TACT (tuned-aperture computed tomography). *Dental Traumatology*.

[32] Lynch EJ, Ahlberg KF (1984). Bilateral idiopathic root resorption of upper first premolars. *International Endodontic Journal*.

[33] Allen AL, Gutmann JL (1977). Internal root resorption after vital root resection. *Journal of Endodontics*.

[34] Trope M, Chivian N, Cohen ST, Burns R (1994). Root resorption. *Pathways of the Pulp*.

[35] Heithersay GS (1985). Clinical endodontic and surgical management of tooth and associated bone resorption. *International Endodontic Journal*.

[36] Culbreath TE, Davis GM, West NM, Jackson A (2000). Treating internal resorption using a syringeable composite resin. *Journal of the American Dental Association*.

[37] Jacobovitz M, de Lima RKP (2008). Treatment of inflammatory internal root resorption with mineral trioxide aggregate: a case report. *International Endodontic Journal*.

[38] Eidelman E, Rotstein I, Gazit D (1997). Internal coronal resorption of a permanent molar: a conservative approach for treatment. *The Journal of Clinical Pediatric Dentistry*.

